# Antithyroid Drug-Induced Agranulocytosis: A Case Report

**DOI:** 10.7759/cureus.48264

**Published:** 2023-11-04

**Authors:** Micaela MacKay, Madison C Clewis, Patrick Sweet

**Affiliations:** 1 Internal Medicine, University of Texas Medical Branch, Galveston, USA

**Keywords:** graves' disease, drug-induced agranulocytosis, antithyroid drugs, agranulocytosis, drug-reaction, methimazole

## Abstract

Agranulocytosis is a rare but life-threatening complication of methimazole and propylthiouracil, antithyroid drugs (ATDs) prescribed for the treatment of hyperthyroidism. We report the case of a 41-year-old female who presented to our institution with complaints of fevers, chills, sore throat, myalgias, and generalized weakness one month after treatment initiation with methimazole. A complete blood count at admission revealed agranulocytosis with an absolute neutrophil count of 0/μl. After discontinuation of the medication, she was treated with granulocyte-colony stimulating factor and intravenous broad-spectrum antibiotics, which improved her condition on day seven of hospitalization.

Although agranulocytosis is a rare complication of antithyroid drugs, providers must maintain a high index of clinical suspicion as prompt diagnosis and treatment are essential. After the diagnosis is confirmed with an absolute neutrophil count <500/μl, management involves discontinuation of the offending agent and initiation of intravenous broad-spectrum antibiotics. Granulocyte-colony stimulating factor, commonly employed in addition to antibiotics, is a controversial treatment option and more research demonstrating its efficacy is necessitated. Preventing mortality associated with antithyroid drug-induced agranulocytosis is achieved through patient education at the time of ATD initiation.

## Introduction

Antithyroid drugs (ATDs) are commonly prescribed for the treatment of hyperthyroidism as they effectively reduce thyroid hormone levels while avoiding the permanent thyroid dysfunction induced by invasive treatment alternatives [1]. Methimazole and propylthiouracil are ATDs that reduce circulating thyroid hormone levels through inhibition of thyroid iodide peroxidase, resulting in decreased thyroid hormone biosynthesis. Drug-induced agranulocytosis, defined as an absolute neutrophil count (ANC) <500/μl of blood, is an extremely rare complication estimated to occur in 0.2-0.5% of patients prescribed ATDs for Graves’ disease [2]. Although the pathophysiology is poorly understood, proposed mechanisms include immune response activation by reactive ATD metabolites or antibody formation against granulocytes. 

The clinical presentation of ATD-induced agranulocytosis is variable, and the diagnosis should be considered in any patient prescribed ATDs with infectious symptoms. Patients most commonly present with a high fever and sore throat within three months of initiating treatment with ATDs. Diagnosis requires an ANC <500/μl during treatment with an ATD or within seven days of previous exposure. Exclusion criteria include: history of congenital or immune neutropenia; recent infectious disease; underlying hematological disease; and recent chemotherapy, radiotherapy, or immunotherapy. 

Management steps include (1) discontinuation of the offending medication, (2) exclusion of associated infections, and (3) initiation of intravenous (IV) broad-spectrum antibiotics. Although granulocyte-colony stimulating factor (G-CSF) is commonly used to treat ATD-induced agranulocytosis, the literature demonstrating its efficacy is limited, and some studies suggest the therapy may not shorten the time to ANC normalization [3].

ATD-induced agranulocytosis resolves in 10 days on average. Patient education at the time of ATD initiation is recommended to prevent mortality associated with this rare complication. Periodic screening measurements of the ANC are not recommended due to their limited utility in diagnosing the abrupt-onset agranulocytosis induced by ATDs.

We report the case of a female patient with Graves' disease who developed agranulocytosis one month after treatment initiation with methimazole. The patient was treated with G-CSF and IV broad-spectrum antibiotics, with eventual normalization of her ANC.

## Case presentation

A 41-year-old woman presented to our institution with a six-day history of fevers, chills, sore throat, myalgias, and generalized weakness. The patient's symptoms were unimproved despite completing a five-day course of amoxicillin-clavulanate. Pertinent medical history included a recent diagnosis of Graves’ disease, for which she had been prescribed methimazole (10 mg daily) one month prior. 

Vital signs at admission were significant for a pulse of 103 beats per minute and temperature of 103.1°F. A general examination revealed enlarged tonsils and tachycardia but was otherwise unremarkable. Laboratory testing at admission revealed severe leukopenia and neutropenia.

The patient was treated with IV broad-spectrum antibiotics and G-CSF for ATD-induced agranulocytosis, a diagnosis supported by her severe neutropenia one month after ATD initiation. Her tachycardia, thyroid function tests, and recent discontinuation of methimazole suggested thyrotoxicosis, for which she was administered propranolol, saturated solutions of potassium iodide, and cholestyramine. 

Treatment with IV antibiotic therapy and G-CSF continued over the following days, but the patient showed minimal clinical improvement. She remained intermittently febrile and tachycardic, with average heart rates ranging from 102 to 120 bpm. Her white blood cell count remained well below the normal range. Despite an extensive workup including blood and throat cultures, a respiratory pathogen panel, COVID-19 testing, influenza PCR, urinalysis, and chest x-ray, no infectious source of neutropenic fever was identified.

On the seventh day of admission, the patient's condition improved drastically. Her white blood cell count increased to 2.78 from 0.95 at admission. Vital signs, including pulse and temperature, were within normal limits (Table 1). 

**Table 1:** Daily vital signs. Pulse (bpm): average daily pulse (beats per minute); Temp (°F): average daily temperature (degrees Fahrenheit); Tmax (°F): maximum documented temperature (degrees Fahrenheit); MAP (mmHg): mean arterial pressure (millimeters of mercury).

	Pulse (bpm)	Temp (°F)	Tmax (°F)	MAP (mmHg)
Admission (Day 1)	112	100.9	103.1	76
Day 2	102	100.1	102.3	82
Day 3	104	100.7	102.4	86
Day 4	113	101.3	103.0	80
Day 5	120	100.1	102.7	73
Day 6	113	100.1	102.4	81
Day 7	89	98.2	100.6	78
Day 8	82	97.5	98.0	81
Discharge (Day 9)	78	97.7	97.2	76

After two additional days of continued improvement, the patient was discharged. On the day of discharge, she had remained afebrile for 48 hours, and her agranulocytosis had resolved (Table 2). She underwent outpatient thyroidectomy for definitive treatment of Graves’ disease, and laboratory investigations since discharge continue to demonstrate white blood cell counts within normal limits.

**Table 2:** CBC and TFTs at admission (2A) and discharge (2B). CBC: complete blood count; TFTs: thyroid function tests; WBC: white blood cells; RBC: red blood cells; HGB: hemoglobin; HCT: hematocrit; MCV: mean corpuscular volume; PLT: platelet count; ANC: absolute neutrophil count; TSH: thyroid-stimulating hormone; Free T4: free thyroxine.

Component (ref range and units)	Result at admission (2A)	Result at discharge (2B)
WBC (4.30-11.10 × 10^3^/µL)	0.95	24.02
RBC (3.93-5.25 × 10^6^/µL)	4.18	4.19
HGB (11.6-15.0 g/dL)	9.4	9.5
HCT (35.7-45.2%)	29.4	29.8
MCV (80.6-95.5 fL)	70.3	71.1
PLT (166-358 × 10^3^/µL)	281	498
ANC (1.88-7.09 × 10^3^/uL)	0 (Comment: no neutrophils counted)	17.1
TSH (0.45-4.70 mIU/L)	<0.02	-
Free T4 (0.78-2.20 ng/dL)	5.26	2.44

## Discussion

Agranulocytosis, characterized by an ANC of less than 500/μl, is a rare but serious complication of ATDs [4]. The pathophysiology is thought to involve either immune-mediated mechanisms triggered by the drug's reactive metabolites or the formation of antibodies against granulocytes [5]. The severe reduction in ANC increases susceptibility to bacterial infections, which may become invasive and life-threatening.

Early diagnosis is essential in ATD-induced agranulocytosis to prevent the progression of infection and associated mortality. The most common symptoms at presentation are fever (>90% of cases) and sore throat (around 80% of cases) [6]. Initial clinical diagnoses commonly include pharyngitis, tonsillitis, and pneumonia. The similarity in the presentations of ATD-induced agranulocytosis and common infectious illnesses may result in missed diagnoses and treatment delays, as seen in our patient's case. A week before presenting to our institution, the patient sought medical attention but was diagnosed with pharyngitis and prescribed oral antibiotics. Failure to exclude the diagnosis of ATD-induced agranulocytosis by obtaining a CBC at that time resulted in treatment delay and worsening of the patient's condition. ATD-induced agranulocytosis should be considered and excluded in all patients on ATDs presenting with infectious symptoms.

Management of ATD-induced agranulocytosis involves ATD discontinuation to normalize the ANC and the initiation of IV broad-spectrum antibiotics to prevent invasive bacterial infection. Definitive treatment options, including thyroidectomy and radioactive iodine ablation, should be considered for the management of hyperthyroidism upon improvement of agranulocytosis. G-CSF is commonly employed to aid in the normalization of the ANC by accelerating neutrophil recovery, but its use is controversial. Although some studies suggest G-CSF may effectively shorten the recovery duration of ATD-induced agranulocytosis, these studies found a mean difference of only 3.16 days among Asian cohorts, and efficacy was not demonstrated in South American or European cohorts [7]. Additionally, retrospective and prospective studies have found the therapy does not significantly improve the recovery time of agranulocytosis induced by ATDs [3,8]. Our patient's ANC normalized on G-CSF treatment day seven, potentially suggesting a lack of efficacy of the therapy as ATD-induced agranulocytosis often improves within a week of ATD discontinuation without intervention. Further research is needed to determine the efficacy of G-CSF for the treatment of ATD-induced agranulocytosis.

Patient education at the time of ATD initiation is essential to prevent the morbidity and mortality associated with ATD-induced agranulocytosis. Guidelines recommend physicians educate patients on common symptoms of agranulocytosis, including fever and sore throat, and instruct them to seek medical advice urgently upon symptom development. Despite this, studies suggest inadequate education, as more than 60% of patients receiving ATDs are not aware of the symptoms of agranulocytosis [9]. Improving patient education on ATD-induced agranulocytosis is crucial to prevent mortality associated with the condition and improve outcomes.

## Conclusions

This case report highlights the clinical presentation, diagnosis, and management of ATD-induced agranulocytosis. Clinicians should maintain a high index of suspicion for this rare but life-threatening complication of ATDs in any patient presenting with infectious symptoms. Management of ATD-induced agranulocytosis involves prompt discontinuation of the offending medication and the initiation of IV broad-spectrum antibiotics. Further research evaluating the efficacy of G-CSF in shortening the duration of agranulocytosis is necessitated. Preventing mortality associated with ATD-induced agranulocytosis is achieved through patient education at the time of ATD initiation.
